# Three-Year Outcomes in Kidney Transplant Patients Randomized to Steroid-Free Immunosuppression or Steroid Withdrawal, with Enteric-Coated Mycophenolate Sodium and Cyclosporine: The Infinity Study

**DOI:** 10.1155/2014/171898

**Published:** 2014-03-05

**Authors:** A. Thierry, G. Mourad, M. Büchler, G. Choukroun, O. Toupance, N. Kamar, F. Villemain, Y. Le Meur, C. Legendre, P. Merville, M. Kessler, A.-E. Heng, B. Moulin, S. Queré, F. Di Giambattista, A. Lecuyer, G. Touchard

**Affiliations:** ^1^Service de Néphrologie, Hémodialyse et Transplantation Rénale, Hôpital La Milétrie, et INSERM U927, CHU, 86021 Poitiers, France; ^2^Service de Néphrologie et Transplantation Rénale, Hôpital Lapeyronie, 371 rue du Doyen Gaston Giraud, 34295 Montpellier, France; ^3^Service de Transplantation Rénale, CHU Bretonneau, 2, Boulevard Tonnellé, 37044 Tours Cedex, France; ^4^Service de Néphrologie, Médecine Interne, Dialyse, Transplantation Rénale et Réanimation, Hôpital Sud, Avenue Laënnec, 80054 Amiens Cedex 1, France; ^5^CHU Amiens et INSERM ERI-12, Université Jules Verne Picardie, Chemin du Thil, 80000 Amiens, France; ^6^Service de Néphrologie et Transplantation Rénale, Hôpital Maison Blanche, 45 rue Cognacq Jay, 51092 Reims Cedex, France; ^7^Service de Néphrologie-Transplantation, Hôpital de Rangueil, 1 rue Jean Poulhès, 31403 Toulouse Cedex 4, France; ^8^Service de Néphrologie, CHU Angers, 4 rue Larrey, 49033 Angers, France; ^9^Service de Néphrologie et Transplantation Rénale, Hôpital La Cavale Blanche, Boulevard Tanguy Prigent, 29200 Brest, France; ^10^Service de Néphrologie et Transplantation Rénale, Hôpital Necker, 161 rue de Sèvres, 75015 Paris, France; ^11^Service de Néphrologie et Transplantation Rénale, Hôpital Pellegrin, Place Amélie Raba Léon, 33076 Bordeaux, France; ^12^Service de Néphrologie et Transplantation Rénale, CHU de Nancy, rue du Morvan, 54511 Vandoeuvre les Nancy, France; ^13^Service de Néphrologie, Hôpital Gabriel Montpied, rue Montalembert, 63003 Clermont Ferrand Cedex 1, France; ^14^Clinique Médicale B, Hôpital Civil, 1 Place de l'Hôpital, 67091 Strasbourg Cedex, France; ^15^Unité Immuno-Transplantation, Novartis Pharma SAS, rue Lionel Terray, 92500 Rueil-Malmaison, France

## Abstract

In a six-month, multicenter, open-label trial, *de novo* kidney transplant recipients at low immunological risk were randomized to steroid avoidance or steroid withdrawal with IL-2 receptor antibody (IL-2RA) induction, enteric-coated mycophenolate sodium (EC-MPS: 2160 mg/day to week 6, 1440 mg/day thereafter), and cyclosporine. Results from a 30-month observational follow-up study are presented. Of 166 patients who completed the core study on treatment, 131 entered the follow-up study (70 steroid avoidance, 61 steroid withdrawal). The primary efficacy endpoint of treatment failure (clinical biopsy-proven acute rejection (BPAR) graft loss, death, or loss to follow-up) occurred in 21.4% (95% CI 11.8–31.0%) of steroid avoidance patients and 16.4% (95% CI 7.1–25.7%) of steroid withdrawal patients by month 36 (*P* = 0.46). BPAR had occurred in 20.0% and 11.5%, respectively (*P* = 0.19). The incidence of adverse events with a suspected relation to steroids during months 6–36 was 22.9% versus 37.1% (*P* = 0.062). By month 36, 32.4% and 51.7% of patients in the steroid avoidance and steroid withdrawal groups, respectively, were receiving oral steroids. In conclusion, IL-2RA induction with early intensified EC-MPS dosing and CNI therapy in *de novo* kidney transplant patients at low immunological risk may achieve similar three-year efficacy regardless of whether oral steroids are withheld for at least three months.

## 1. Introduction

Steroid avoidance is now frequently attempted in *de novo* kidney transplant recipients at low immunological risk [1] to prevent the long-term complications associated with maintenance steroid therapy. Patients may be given only intravenous steroid administration at the time of transplant with no oral steroids at all or receive oral steroids for only a few days after transplant before withdrawal. A regimen that includes induction therapy, a calcineurin inhibitor (CNI), and mycophenolic acid (MPA) in patients who are not at high immunological risk appears to support such an approach without loss of efficacy [2–6], but minor increases in the rate of acute rejection have been reported [2]. In *de novo* kidney transplant patients receiving a standard regimen of steroids, use of an intensified MPA dosing regimen in the early after transplant period when the risk of rejection is the highest has been shown to reduce rejection [7, 8] prompting interest in early intensified MPA therapy when implementing a steroid avoidance strategy. The randomized, multicenter DOMINOS study compared a regimen in which patients were given no oral steroids versus a regimen of standard oral steroids for at least three months, followed by steroid withdrawal where appropriate, in *de novo* low-risk kidney transplant patients receiving an induction by interleukin 2 receptor (IL-2R) inhibitor, cyclosporine (CsA), and early intensified enteric-coated mycophenolate sodium (EC-MPS) dosing to week 6 after transplant [9]. Results showed that the absence of oral steroid therapy in this setting did not compromise efficacy at six months after transplantation [9], but the long-term effect of this regimen is of particular interest. Steroid-related adverse events which unfavorably affect cardiovascular risk, notably dyslipidemia, hyperglycemia, hypertension, and weight gain [10], are of special concern in kidney transplant patients in view of the high rate of cardiovascular-related mortality in this population [11–13]. However, the major randomized trials of steroid avoidance in patients receiving CNI therapy with MPA have followed patients to only six [4], 12 [2, 3, 6] or 24 months [5] after transplant and have reported mixed results concerning the effect of a steroid avoidance regimen on metabolic complications. Longer-term data on the efficacy and safety implications of a steroid avoidance MPA-based immunosuppressive strategy in this setting are of key clinical interest.

## 2. Methods

### 2.1. Study Design and Conduct

Patients who completed the multicenter, randomized, parallel-group, open-label, six-month DOMINOS trial [9] and were receiving EC-MPS and CsA with or without steroids were eligible to enter a further 30-month observational study (INFINITY) during which immunosuppression was administered according to local protocol. The INFINITY study was conducted during October 2007 to October 2011 at all 14 of the French transplant centers that took part in the DOMINOS trial.

The study was undertaken in accordance with the Declaration of Helsinki and the ICH Harmonized Tripartite Guidelines for Good Clinical Practice. All patients provided written informed consent for participation following ethical approval from the Comité de Protection des Personnes (Poitiers, France).

### 2.2. Eligibility Criteria

The DOMINOS study recruited male or female patients aged 18–70 years who received first or second kidney transplant from a deceased, living-related, or living-unrelated donor with panel reactive antibodies (PRA) below 20% at the last pretransplant assessment. Patients were excluded if they received multiorgan transplant (including two kidneys) or had received previous nonrenal transplant, if graft donation was after cardiac death or if the cold ischemia time was more than 36 hours. Patients who completed the DOMINOS trial were eligible to enter the INFINITY study if they remained on EC-MPS and CsA with or without steroid therapy and the investigator planned to continue this regimen.

### 2.3. Immunosuppression

Patients in both treatment arms received a perioperative dose of 500 mg intravenous methylprednisolone, after which patients randomized to the steroid avoidance group received no further steroids unless clinically mandated. Patients in the control group received oral prednisone at a dose of 1 mg/kg/day (maximum 80 mg/day) for one week tapered to 10 mg/day until month 3 after transplant. After month 3, if the locally read month 3 protocol biopsy was negative for subclinical rejection, the dose was decreased by 2.5 mg per 15 days until steroids were stopped. Steroid therapy was continued at a dose of 10 mg/day if the month 3 biopsy showed subclinical rejection.

All patients received IL-2RA induction (Simulect, Novartis Pharma AG, Basel, Switzerland) according to the local center protocol. EC-MPS (*myfortic*, Novartis Pharma AG, Basel, Switzerland) was administered at a dose of 2160 mg/day in two divided doses to week 6 after transplant, after which the dose was reduced to the standard 1440 mg/day. The dose of CsA (Neoral, Novartis Pharma AG, Basel, Switzerland) was adjusted according to the CsA concentration at two hours after dose (C_2_) based on predefined targets up to month 6 after transplant which were the same in each group. After six months, immunosuppression was according to local protocol.

### 2.4. Primary and Secondary Endpoints

The primary endpoint was the incidence of treatment failure at month 6 (defined as clinical biopsy-proven acute rejection (BPAR) by central review, graft loss, death, or loss to follow-up), was assessed at month 36. Secondary efficacy endpoints included the incidence and severity of BPAR (Banff 1997 classification [14]), graft survival, renal function estimated by abbreviated Modification of Diet in Renal Disease (MDRD) [15], calculated creatinine clearance (Cockcroft-Gault formula [16], adjusted for body surface area), estimated glomerular filtration rate ([eGFR] by Nankivell formula [17]), proteinuria, requirement for steroid therapy, and the cumulative dose of steroids. Safety endpoints included adverse events, serious adverse events, and adverse events considered in the opinion of the investigator to be related to steroid therapy.

### 2.5. Statistical Methods

Data are presented for all patients recruited to the INFINITY study from the time of transplant, that is, including the 6-month period during the randomized DOMINOS study and the subsequent 30 months' observational follow-up. Two-sided 95% confidence intervals (CI) were calculated for the difference in the primary endpoint (treatment failure) between the steroid avoidance and control groups. Kaplan Meier estimates of freedom from treatment failure and BPAR were compared between groups using the log rank test. Comparisons between treatment groups were made using the Chi square test or the Fisher exact test for categorical variables and the Wilcoxon Rank Sum test for continuous variables. All statistical analyses were performed using SAS 8.2 software (SAS Institute, Cary, North Carolina, USA).

## 3. Results

### 3.1. Study Population

**Figure 1:**
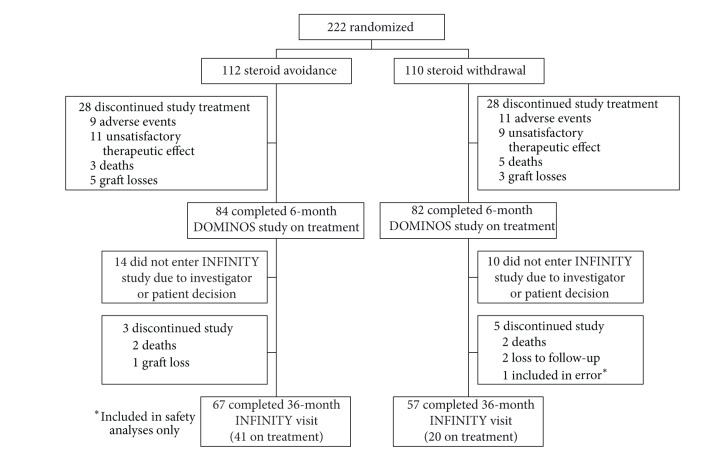
Patient disposition.

In total, 222 patients (112 steroid avoidance, 110 steroid withdrawal) took part in the DOMINOS study, of whom 166 (74.8%; 84 steroid avoidance, 82 steroid withdrawal) completed the study on treatment. In 131 (78.9%) patients, all criteria for entry into the INFINITY study were met, the physician planned to continue the study regimen and agreed to participate in INFINITY, and the patient consented to enter the study (70 steroid avoidance, 61 steroid withdrawal). The 36-month visit was completed by 124 patients, of whom 61 remained on study treatment (Figure 1). One additional patient who had been randomized to the steroid withdrawal group was included inadvertently despite not receiving EC-MPS and CsA at completion of the DOMINOS study and was included only in the safety analyses.

The demographics and baseline characteristics of the patient population were balanced between treatment groups (Table 1). There was a lower proportion of patients with delayed graft function in the steroid avoidance group than the steroid withdrawal group but the difference was not statistically significant (11.4% versus 21.3% group; *P* = 0.12). The INFINITY study population showed no marked differences to the full cohort of patients who took part in the DOMINOS study [9].

### 3.2. Immunosuppression

At month 6 after transplant, 19/70 patients (27.1%) randomized to steroid avoidance were receiving oral steroids. Steroid therapy was introduced in an additional three patients by month 36. In the steroid withdrawal group, 34/61 patients received steroids at month 6 (55.7%) as per protocol (steroids were to be continued if subclinical rejection was observed on the month 3 protocol biopsy); four additional patients discontinued steroids by month 36. Overall, the mean cumulative dose of steroids per patient to month 36 was approximately a third lower in the steroid avoidance group (2467.8 mg versus 3397.7 mg in the steroid withdrawal group; *P* = 0.058) (Table 2).

Of the 126 patients who completed the 36-month visit alive and with a functioning graft, 114 (90.5%) continued to receive MPA therapy. Seventeen patients (13.7%) had switched from CsA to tacrolimus and two patients randomized to the steroid withdrawal group had switched from CNI therapy to a mammalian target of rapamycin (mTOR) inhibitor (Table 2). Data on immunosuppression among the 124 patients who completed the 36-month visit alive and with a functioning graft are shown in Table 2.

### 3.3. Efficacy

**Figure 2:**
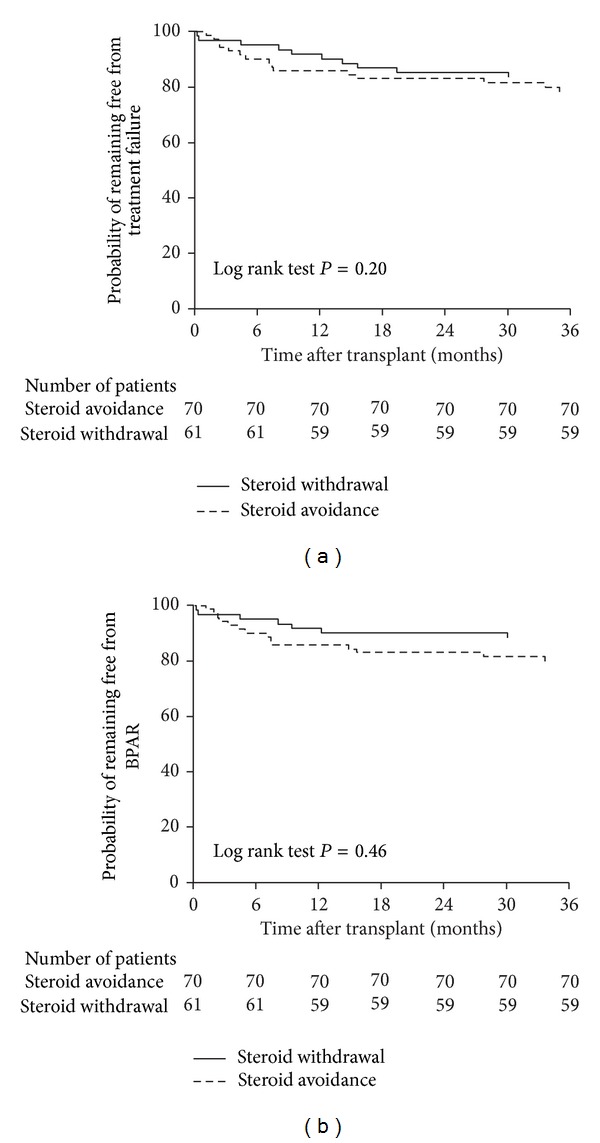
Kaplan-Meier estimates of the probability of remaining free from (a) treatment failure (BPAR (central review), graft loss, death, or loss to follow-up) or (b) BPAR.

The primary efficacy endpoint occurred in 10/70 (14.3%, 95% CI 6.1–22.5%) patients in the steroid avoidance group and 6/61 (9.8%, 95% CI 2.4–17.3%) of the steroid withdrawal group by month 12 after transplant (*P* = 0.44). At month 36 after transplant, the corresponding values were 15/70 (21.4%, 95% CI 11.8–31.0%) and 10/61 (16.4%, 95% CI 7.1–25.7%) (*P* = 0.46). The incidence of BPAR was 20.0% (14/70) in the steroid avoidance group versus 11.5% (7/61) with steroid withdrawal (*P* = 0.19), with the most severe grade of BPAR being classified as grade IA in 9/14 steroid avoidance patients (Table 3). Kaplan-Meier estimates indicated that the probability of remaining free from treatment failure at month 36 was 79.9% and 88.4% in the steroid avoidance and steroid withdrawal groups, respectively (*P* = 0.20, log rank test) (Figure 2(a)). The corresponding values for BPAR were 78.4% and 83.6% (*P* = 0.46, log rank test) (Figure 2(b)).

Between month 6 and month 36, the rate of treatment failure was 12.9% (9/70) in the steroid avoidance group and 13.1% (8/61) in the steroid withdrawal group. BPAR occurred in 7 and 4 patients, respectively. All episodes were graded IA or IB except one episode in the steroid withdrawal group which was graded IIA (Table 3). One graft was lost in the steroid avoidance group due to a transplantectomy for perirenal hematoma compression. Two patients died in each group, due to metastatic bronchial carcinoma and unknown causes in the steroid avoidance group, and epidermoid cancer and multiorgan failure syndrome in the steroid withdrawal group.

**Figure 3:**
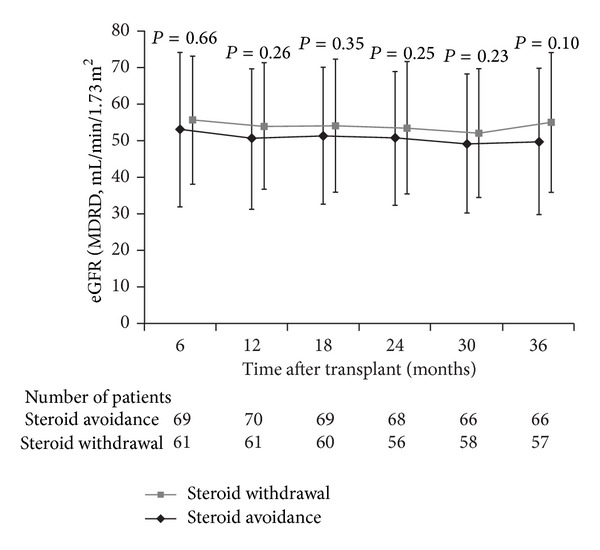
Estimated GFR (MDRD) during months 6 to 36. Values are shown as mean (SD). GFR: glomerular filtration rate; MDRD: Modification of Diet in Renal Disease; SD: standard deviation.

Renal function did not differ between the two groups during the study. Mean (SD) eGFR (MDRD) was not significantly different at month 6 after transplant (steroid avoidance 53.2 [17.6] mL/min/1.73 m^2^ versus steroid withdrawal 55.8 [21.1] mL/min/1.73 m^2^; *P* = 0.66) and at month 36 (49.9 [19.1] mL/min/1.73 m^2^ versus 55.1 [20.0] mL/min/1.73 m^2^; *P* = 0.10) (Figure 3). Similar findings were observed when renal function was assessed by calculated creatinine clearance (Cockcroft-Gault formula) or when eGFR was estimated by the Nankivell formula (data not shown). Mean proteinuria was also similar between groups at month 6 (0.3 ± 0.4 g/mmol in the steroid avoidance group versus 0.4 ± 0.5 g/mmol in the steroid withdrawal groups; *P* = 0.56) and month 36 (0.5 ± 1.0 g/mmol versus 0.4 ± 0.5 g/mmol; *P* = 0.68).

### 3.4. Adverse Events

Almost all patients reported at least one adverse event during the follow-up study (months 6 to 36), with no difference between treatment groups (steroid avoidance 69/70 (98.6%), steroid withdrawal 60/62 (96.8%); *P* = 0.60). The most frequent adverse events were dyslipidemia, diarrhea, peripheral edema, and urinary tract infection (14.3%, 18.6%, 18.6%, and 12.9% in the steroid avoidance group, respectively, and 24.2%, 19.4%, 16.1%, and 16.1% in the steroid withdrawal group), the incidence of which did not differ significantly between groups. Serious adverse events were reported in 34 steroid avoidance patients and 33 steroid withdrawal patients (48.6% versus 53.2%, *P* = 0.52). The incidence of adverse events with a suspected relation to steroids during months 6–36 was 22.9% (16/70) and 37.1% (23/62) in the steroid avoidance and steroid withdrawal groups, respectively (*P* = 0.062). The corresponding values for serious adverse events with a suspected relation to steroids during months 6–36 were 8.6% (6/70) and 6.5% (4/62) in the steroid avoidance and steroid withdrawal groups, respectively (*P* = 0.75).

Seven steroid avoidance patients and nine steroid withdrawal patients discontinued study drug due to adverse events.

The incidence of infections during months 6 to 36 was 64.3% (*n* = 45) in the steroid avoidance group and 77.4% (*n* = 48) in the steroid withdrawal group. The significant difference in cytomegalovirus (CMV) infection reported as an adverse event in the DOMINOS study population at month 6 (12.5% versus 22.7%, *P* = 0.045) became nonsignificant during months 6–36 (10.0% versus 6.5%, *P* = 0.48).

The proportion of patients receiving antihypertensive treatment, lipid-lowering treatment or hypoglycemic treatment at month 36 was 94.9%, 69.2%, and 15.4%, respectively, in the steroid avoidance group compared to 88.5%, 73.6%, and 13.8% in the steroid withdrawal group. Body mass index (BMI) at time of transplant was 24.9 ± 3.7 kg/m^2^ versus 25.3 ± 4.7 kg/m^2^ in the steroid avoidance and steroid withdrawal arms, respectively (*P* = 0.85), and 25.6 ± 4.3 kg/m^2^ versus 27.5 ± 6.1 kg/m^2^ at month 36 (*P* = 0.13). The increase in BMI was not significantly different between randomized groups (mean difference 0.82 kg/m^2^; 95% CI −0.33, 1.98; 0.16). However, when the change in BMI was compared between those patients who remained steroid free throughout follow-up (*n* = 35) and those who received steroids at some point (*n* = 75) and for whom BMI data were available at baseline and month 36, the increase was significantly lower in the steroid-free cohort (0.52 ± 0.50 versus 1.97 ± 0.34 in steroid-treated patients, *P* = 0.019).

## 4. Discussion

Maintenance steroid therapy remains widespread following kidney transplantation, both in recent clinical trials [18, 19] and in daily practice, although the shift towards steroid avoidance or sparing continues to gather momentum. A meta-analysis by Pascual et al. has confirmed that steroid avoidance or withdrawal is possible in kidney transplantation [13] but the optimal timing for steroid-free immunosuppression has not been clearly defined. Results from this observational follow-up trial suggest that early intensified EC-MPS dosing with CNI therapy and IL-2RA induction may permit long-term steroid avoidance in a substantial proportion of low-risk kidney transplant recipients without compromising efficacy to three years after transplant. There was no statistically significant difference for graft survival between patients who did or did not initially receive steroid therapy.

In the DOMINOS study population, BPAR was not more frequent in the steroid avoidance group at month 3 or month 6 versus the steroid withdrawal group, and no episodes of BPAR in the steroid avoidance group were more severe than grade IIA [9]. In the INFINITY study, the incidence of BPAR at month 36 was numerically higher in the steroid avoidance group (20.0% versus 11.5% with steroid withdrawal), as reported elsewhere [2]. However, the absolute rate of BPAR was low in both arms with no episode of rejection graded higher than IIA in the steroid avoidance group, and the difference between groups was not significant. Several aspects of the study protocol are likely to have contributed to the preservation of efficacy despite the steroid-free regimen. Patients with high PRA levels or an extended cold ischemia time were excluded. 92.4% of the population is Caucasian. The feasibility of this type of steroid avoidance regimen in higher-risk individuals, such as patients with donor specific antibodies, African-American recipients, or those receiving a marginal graft from an extended criteria donor, is questionable. An intensified regimen of EC-MPS was administered during the first six weeks after transplantation. Such a regimen has previously been shown to reduce the risk of BPAR when administered to patients receiving CsA and IL-2RA induction [7]. Moreover, protocol biopsies at three months after transplantation ensured detection of subclinical pathology, permitting reevaluation of the immunosuppressive regimen and reintroduction of steroids or other revisions if necessary. This approach improves the security of steroid avoidance. An alternative option may be to continue steroids indefinitely and withdraw long-term MPA therapy although comparative data from randomized controlled trials is relatively sparse [20].

In terms of renal function, mean creatinine clearance at month 36 was 44.7 mL/min/1.73 m^2^. Comparison of renal function between our population and other steroid avoidance studies is hampered by the fact that eGFR has not always been reported [3, 5] and that, where available, values extend only to month 6 [4] or month 12 [2], but published values at those time points are broadly similar to those observed in our study. Moreover, there was no significant difference in renal function between the two treatment groups at any time point. The potential concern that steroid avoidance could ultimately lead to chronic rejection seems to be addressed by the finding that renal function, proteinuria, and the reported incidence of chronic rejection were similar in both arms to 36 months after transplant. It would also have been interesting to assess the presence of donor specific antibodies to evaluate whether this was promoted by steroid withdrawal, but the study protocol did not address this question.

Other aspects of the study merit discussion. The INFINITY study was observational in design, with investigators free to manage patients according to local protocol after month 6 after transplant. Nevertheless, there were relatively few changes to immunosuppressive regimens after month 6, with steroids being introduced in only three patients (4.3%) in the steroid avoidance arm and being discontinued in only four patients (6.5%) in the steroid withdrawal arm. It is important to note that the trial did not compare true steroid avoidance versus maintenance steroid therapy but instead compared very early steroid discontinuation versus late withdrawal. Since the majority of acute rejection events occur in the first few weeks after kidney transplantation, the study protocol specified that steroids should be tapered and withdrawn in the steroid withdrawal arm if no histological evidence of subclinical rejection was present at month 3. By this protocol, 52.6% of steroid withdrawal patients were steroid-free at month 6. This highlights the difficulty of withdrawing steroids at a later time point (i.e., after three months) compared to an early steroid-free regimen whereby patients received no oral steroids after transplantation. There was also a clinical requirement to introduce steroids before month 6 in 27.1% of patients in the steroid avoidance arm by month 6 (most frequently in response to suspected or confirmed acute rejection). Consequently, there was considerable overlap in steroid administration between the two groups during months 6 to 36. Nevertheless the mean cumulative steroid dose during months 6 to 36 in the cohort randomized to steroid avoidance arm was 27% lower than that in the steroid group, a difference that approached statistical significance (*P* = 0.058). Data on adverse events should be interpreted in this context; that is, the greatest difference in steroid exposure between groups occurred during the first three months after transplantation and narrowed thereafter. Thus, although both the metabolic effects of steroids such as hypertension, hyperlipidemia, diabetes mellitus, obesity and endothelial dysfunction, and other effects including osteoporosis and skin atrophy are well recognized [21], it is not unexpected that the between-group differences in the incidence of adverse events and serious adverse events with a suspected relation to steroids which were observed at month 6 [9] became nonsignificant over the period 6–36 months after transplant. Additionally, even the current extended follow-up period of 36 months is probably inadequate to detect the long-term benefit of steroids avoidance, particularly for cardiovascular disease. Recently, 10-year results were reported from a nonrandomized single-center analysis of adult primary kidney transplant patients in whom steroids were discontinued after postoperative day 5 [22]. Patients received rabbit antithymocyte globulin induction therapy, with a CNI (either CsA or tacrolimus) and mycophenolate mofetil or sirolimus. At 10 years after transplant, there was a significant reduction in steroid-related side effects compared to historical controls, with acceptable patient and graft survival. The current randomized, multicenter study confirms that steroid avoidance is also feasible in kidney transplant patients who receive IL-2RA induction, CsA, and early intensified EC-MPS.

In conclusion, these findings suggest that IL-2RA induction with early intensified EC-MPS dosing and CNI therapy in *de novo* kidney transplant patients at low immunological risk is associated with nonsignificant difference in efficacy at three years after transplant whether oral steroids are withheld or administered for at least three months. The robustness of this observation is limited by the fact that approximately a third of “steroid-free” patients resumed steroid therapy and by the protocol-driven withdrawal of steroids in almost half the “steroid-treated” patients. Nevertheless, the results raise questions about the necessity of administering steroids during the first three months after kidney transplantation. The patient population that could obtain the most benefit from avoiding oral steroids remains to be defined in future studies.

## Figures and Tables

**Table 1:** Demographics and baseline characteristics.

	Without initial steroids (*n* = 70)	With initial steroids (*n* = 61)	*P* value
Recipients			
Male gender, *n* (%)	49 (70.0)	35 (57.4)	0.133
Age (years), mean ± SD	52.4 ± 10.0	50.7 ± 11.6	0.496
White race, *n* (%)	65 (92.9)	56 (91.8)	0.120
Body mass index (kg/m^2^), mean ± SD	24.9 ± 3.7	25.9 ± 4.7	0.080
Panel reactive antibodies 0%, *n* (%)	69 (98.6)	61 (100.0)	1.000
Delayed graft function	8 (11.4)	13 (21.3)	0.124
Donors			
Male gender, *n* (%)	43 (61.4)	37 (60.7)	0.928
Age (years), mean ± SD	48.9 (15.4)	46.1 (15.7)	0.276
Deceased donor (heart beating), *n* (%) Living unrelated, *n* (%)	70 (100.0) 0	60 (98.4) 1 (1.6)	0.466
Transplant			
Recipient <60 years, donor <60 years, *n*(%)	50 (71.4)	46 (75.4)	0.607
Recipient ≥60 years, donor ≥60 years, *n* (%)	11 (15.7)	10 (16.4)	0.916
Cold ischemia time (hours), mean ± SD	17.5 (6.0)	16.9 (4.8)	0.937
CMV status, *n* (%)			
D+/R−	21 (30.0)	15 (24.6)	0.489
D+/R+	15 (21.4)	12 (19.7)	0.804
D−/R−	17 (24.3)	21 (34.4)	0.202
D−/R+	17 (24.3)	13 (21.3)	0.686

CMV: cytomegalovirus; SD: standard deviation.

**Table 2:** Immunosuppression at months 6 and 36.

	Month 6			Month 36		
	Steroid avoidance (*n* = 70)	Steroid withdrawal (*n* = 61)	*P* value	Steroid avoidance (*n* = 70)	Steroid withdrawal (*n* = 61)	*P* value
Oral steroids, *n*/*N* (%)	19/70 (27.1)	34/61 (55.7)	0.001	22/68 (32.4)	30/58 (51.7)	0.028
Dose of oral steroids (mg/day)						
Mean ± SD	8.8 ± 1.7	9.6 ± 5.9	0.529	6.2 ± 2.2	5.9 ± 3.2	0.206
Median (range)	10.0 (5.0–10.0)	8.8 (2.5–30.0)		5.5 (1.3–10.0)	5.0 (2.5–20.0)	
Cumulative dose of oral steroids from month 6 (mg)						
Mean ± SD	—	—	—	2467.8 ± 3496	3397.7 ± 3290	0.058
Median (range)	—	—		0 (0–11400)	3710 (0–11995)	
CsA, *n*/*N* (%)	70/70 (100.0)	61/61 (100.0)	—	60/68 (89.6)	45/58 (81.8)	0.333
Tacrolimus, *n*/*N* (%)	—	—		7/68 (10.4)	10/58 (18.2)	
No calcineurin inhibitor, *n*/*N* (%)	—	—		1/68 (1.4)	3/58 (5.2)	
EC-MPS, *n*/*N* (%)	70/70 (100.0)	60/61 (98.4)	0.466	59/68 (93.7)	51/58 (87.9)	0.369
EC-MPS dose (mg/day)						
Mean ± SD	1309 ± 303	1332 ± 259	0.435	1226 ± 335	1161 ± 340	0.242
Median (range)	1440 (180–2160)	1440 (720–1440)		1440 (360–1440)	1440 (540–1440)	
mTOR inhibitor *n*/*N* (%)	—	—	—	0/70	2/58 (3.5)	0.201
Azathioprine *n*/*N* (%)	—	—	—	2/68 (2.9)	3/58 (5.2)	0.661

CsA: cyclosporine; EC-MPS: enteric-coated mycophenolate sodium; mTOR: mammalian target of rapamycin; SD: standard deviation.

Percentages at month 36 are shown using the denominator of all patients with a functioning graft at month 36.

**Table 3:** Efficacy endpoints at month 36, *n* (%).

	Steroid avoidance (*n* = 70)	Steroid withdrawal (*n* = 61)	*P* value
Treatment failure^a^	15 (21.4)	10 (16.4)	0.46^b^
BPAR^c^	14 (20.0)	7 (11.5)	0.19^b^
Grade IA	9	3	
Grade IB	2	1	
Grade IIA	1	3	
Graft loss	1 (1.4)	0 (0.0)	1.00^d^
Death	2 (2.9)	2 (3.3)	1.00^d^
Loss to follow-up	0 (0.0)	2 (3.3)	0.22^d^

BPAR: biopsy-proven acute rejection.

^a^Treatment failure was defined as BPAR (central review), graft loss, death or loss to follow-up. BPAR detected on the 3-month protocol biopsy was excluded.

^b^Chi-squared test.

^c^If a patient experienced more than one episode of BPAR, only the highest rejection grade is shown.

^d^Fisher's exact test.

## References

[1] Luan FL, Steffick DE, Ojo AO (2009). Steroid-free maintenance immunosuppression in kidney transplantation: is it time to consider it as a standard therapy. *Kidney International*.

[2] Vincenti F, Schena FP, Paraskevas S, Hauser IA, Walker RG, Grinyo J (2008). A randomized, multicenter study of steroid avoidance, early steroid withdrawal or standard steroid therapy in kidney transplant recipients. *American Journal of Transplantation*.

[3] Vincenti F, Monaco A, Grinyo J, Kinkhabwala M, Roza A (2003). Multicenter randomized prospective trial of steroid withdrawal in renal transplant recipients receiving basiliximab, cyclosporine microemulsion and mycophenolate mofetil. *American Journal of Transplantation*.

[4] Rostaing L, Cantarovich D, Mourad G (2005). Corticosteroid-free immunosuppression with tacrolimus, mycophenolate mofetil, and daclizumab induction in renal transplantation. *Transplantation*.

[5] Anil Kumar MS, Xiao S-G, Fyfe B (2005). Steroid avoidance in renal transplantation using basiliximab induction, cyclosporine-based immunosuppression and protocol biopsies. *Clinical Transplantation*.

[6] Gheith OA, Nematalla AH, Bakr MA, Refaie A, Shokeir AA, Ghoneim MA (2010). Cost-benefit of steroid avoidance in renal transplant patients: a prospective randomized study. *Scandinavian Journal of Urology and Nephrology*.

[7] Sommerer C, Glander P, Arns W (2011). Safety and efficacy of intensified versus standard dosing regimens of enteric-coated mycophenolate sodium in de novo renal transplant patients. *Transplantation*.

[8] Gourishankar S, Houde I, Keown PA (2010). The CLEAR study: a 5-day, 3-g loading dose of mycophenolate mofetil versus standard 2-g dosing in renal transplantation. *Clinical Journal of the American Society of Nephrology*.

[9] Thierry A, Mourad G, Büchler M (2012). Steroid avoidance with early intensified dosing of enteric-coated mycophenolate sodium: a randomized, multicentre trial in kidney transplant recipients. *Nephrology Dialysis Transplantation*.

[10] Boots JMM, Christiaans MHL, van Hooff JP (2004). Effect of immunosuppressive agents on long-term survival of renal transplant recipients: focus on the cardiovascular risk. *Drugs*.

[11] Pilmore H, Dent H, Chang S, McDonald SP, Chadban SJ (2010). Reduction in cardiovascular death after kidney transplantation. *Transplantation*.

[12] Berthoux F, Mariat C (2010). Cardiovascular death after renal transplantation remains the first cause despite significant quantitative and qualitative changes. *Transplantation*.

[13] Pascual J, Zamora J, Galeano C, Royuela A, Quereda C (2009). Steroid avoidance or withdrawal for kidney transplant recipients. *Cochrane Database of Systematic Reviews*.

[14] Cendales LC, Kanitakis J, Schneeberger S (2008). The Banff 2007 working classification of skin-containing composite tissue allograft pathology. *American Journal of Transplantation*.

[15] Poggio ED, Wang X, Weinstein DM (2006). Assessing glomerular filtration rate by estimation equations in kidney transplant recipients. *American Journal of Transplantation*.

[16] Cockcroft DW, Gault MH (1976). Prediction of creatinine clearance from serum creatinine. *Nephron*.

[17] Nankivell BJ, Gruenewald SM, Allen RDM, Chapman JR (1995). Predicting glomerular filtration rate after kidney transplantation. *Transplantation*.

[18] Ekberg H, Tedesco-Silva H, Demirbas A (2007). Reduced exposure to calcineurin inhibitors in renal transplantation. *The New England Journal of Medicine*.

[19] Silva HT, Cibrik D, Johnston T (2010). Everolimus plus reduced-exposure CsA versus mycophenolic acid plus standard-exposure CsA in renal-transplant recipients. *American Journal of Transplantation*.

[20] Pascual J, Van Hooff JP, Salmela K, Lang P, Rigotti P, Budde K (2006). Three-year observational follow-up of a multicenter, randomized trial on tacrolimus-based therapy with withdrawal of steroids or mycophenolate mofetil after renal transplant. *Transplantation*.

[21] Poetker DM, Reh DD (2010). A comprehensive review of the adverse effects of systemic corticosteroids. *Otolaryngologic Clinics of North America*.

[22] Rizzari MD, Suszynski TM, Gillingham KJ (2012). Ten-year outcome after rapid discontinuation of prednisone in adult primary kidney transplantation. *Clinical Journal of the American Society of Nephrology*.

